# Unilateral crosstalk cancellation via bone conduction: Methods and evaluation

**DOI:** 10.1016/j.mex.2023.102394

**Published:** 2023-10-02

**Authors:** Irwansyah, Sho Otsuka, Seiji Nakagawa

**Affiliations:** a Center for Frontier Medical Engineering, Chiba University, Chiba, Japan; b Med-Tech Link Center, Chiba University, Chiba, Japan; c Department of Medical Engineering, Graduate School of Science and Engineering, Chiba University, Chiba, Japan

**Keywords:** Crosstalk cancellation, Bone conduction, Adaptive algorithm, Hearing threshold, Bone transducer, Error Sensor-Based Crosstalk Reduction in Bone Conduction

## Abstract

Bone conduction hearing aids (BCHAs) offer an alternative solution for individuals with outer or middle ear issues who cannot benefit from traditional air conduction hearing aids. However, the phenomenon of “crosstalk,” where sound intended for one ear is mistakenly transmitted to the other ear through bone conduction, presents a challenge. This unintended transmission may limit the benefits of binaural hearing that can be achieved using two BCHAs, such as accurately detecting a sound source's direction. In this article, we present a method to suppress “crosstalk” within the human head using an adaptive algorithm to control two audiometric bone transducers.

• Our method involves positioning an error sensor at a location considered close to the cochlea, such as the ear canal or the mastoid, and utilizing an adaptive algorithm to estimate the crosstalk compensation filter. This filter generates an anti-signal, which is then transmitted to one of the two transducers, effectively cancelling the crosstalk.
• To verify whether the crosstalk cancellation reaches the cochlea in the inner ear, we provide a procedure for measuring hearing thresholds with and without crosstalk cancellation. This acts as a subjective measure of the efficacy of our crosstalk cancellation method.

By leveraging an adaptive algorithm, this approach provides personalized cancellation and has the potential to enhance the performance of binaural BCHAs.

## Graphical abstract

**Figure:**
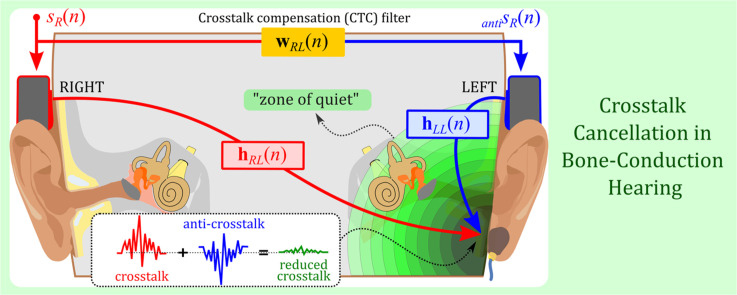

Specifications Table

**Table:** 

Subject area:	Medicine and Dentistry
More specific subject area:	Audiology
Name of your method:	Error Sensor-Based Crosstalk Reduction in Bone Conduction.
Name and reference of original method:	Irwansyah, S. Otsuka, and S. Nakagawa, “Improved Low-Frequency Crosstalk Cancellation in Bone Conduction Using Bone Transducers and Probe Microphone,” in *IEEE Access*, 10 (2022) 79,201–79,212. https://doi.org/10.1109/ACCESS.2022.3194171
Resource availability:	N.A.

**Method details**

## Introduction

### Brief introduction to the problem of “crosstalk” in bone conduction

**Fig. 1:**
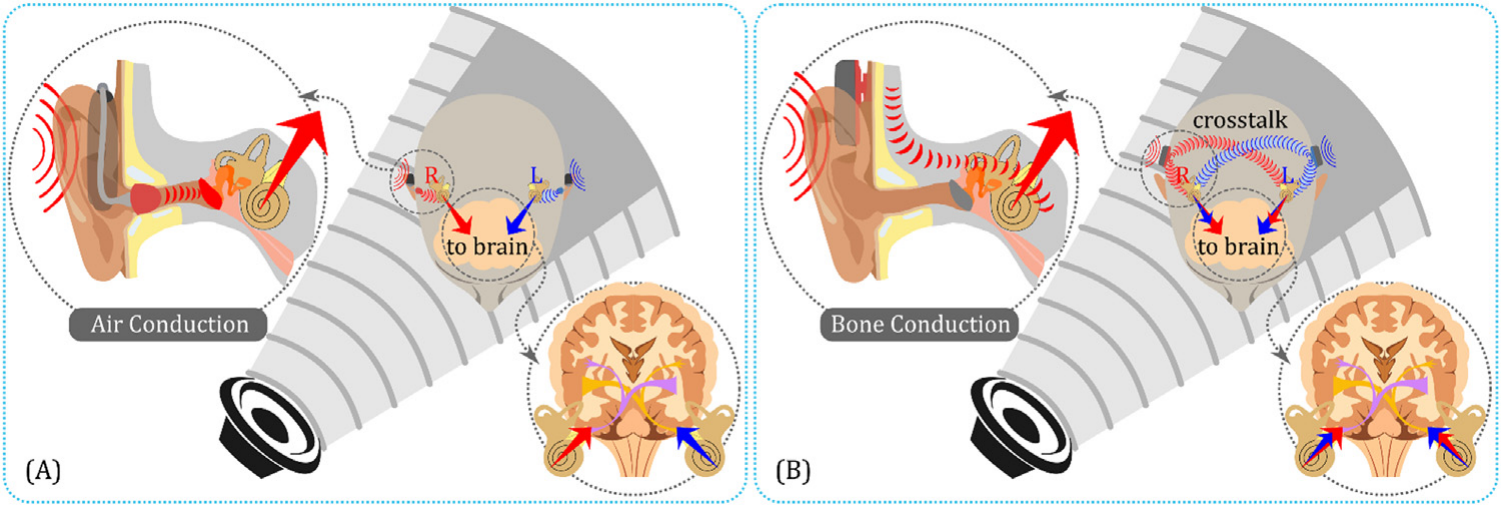
(a) Binaural hearing with air conduction. (b) Binaural hearing via bone conduction, illustrating “crosstalk,” where sounds from each transducer reach both cochleae, potentially limiting the central auditory system's ability to utilize binaural cues.

Sound can reach the cochlea in the inner ear via two mechanisms: air conduction (AC) and bone conduction (BC). AC sound enters the ear canal, vibrates the eardrum, and sets the ossicles in motion, ultimately transmitting the sound to the cochlea (Fig. 1a). In contrast, BC sound bypasses the eardrum and ossicles, directly stimulating the cochlea (Fig. 1b). This direct stimulation provides a significant benefit: For individuals with conductive hearing loss (CHL), which affects the outer or middle ear but leaves the cochlea intact, BC hearing aids (BCHAs) can serve as a viable alternative to AC hearing aids. Despite the advantages of binaural hearing, only a small fraction (6 %) of individuals requiring BCHAs use two devices [1]. This is strikingly different from the situation with AC hearing aids, where the majority (70 %) typically choose two devices.

One of the limitations of BC hearing aids that can significantly impact the primary binaural cues of interaural phase and level differences (IPD and ILD) is “crosstalk,” or undesirable BC transmission. This phenomenon occurs when BC sounds from the left and right sides get mixed at both cochleae (Fig. 1b), resulting in the potential impairment of binaural processing in the central auditory system. This can subsequently decrease the ability to localize sound sources, especially at extreme angles [2], [3], [4]. Psychoacoustic tests using a pair of audiometric bone transducers have demonstrated that sound localization accuracy is typically limited to within 30° [2] or 45° [3,4]. Moreover, when compared with normal AC hearing, binaural hearing with BC presents less benefit in speech-based tests [5]. These limitations are generally attributed to “crosstalk.”

### On the current method of implementing crosstalk cancellation on the living human head

In the context of binaural sound reproduction, the “crosstalk” issue encountered in BC is analogous to the challenges faced when using a pair of loudspeakers [6,7]. With headphones, binaural sound can be delivered without any crosstalk; however, when using two loudspeakers, a crosstalk cancellation method is necessary to counteract the sound from the opposite speaker that reaches the listener's ears. Typically, this is achieved by placing binaural microphones in the listener's ears to measure the crosstalk, and then generating a compensation filter based on these measurements. Applying this strategy to BC, however, may present a unique challenge as the target site for cancellation is the cochlea, which is inaccessible for sensor placement [8].

In an effort to address the challenge of “crosstalk,” McLeod and Culling developed a technique based on psychoacoustics to measure the phase and level of BC sounds reaching each cochlea. This approach made use of a pair of bone transducers alongside two insert earphones [9]. Later modifications led to the development of the “two-BT” method, which relied solely on bone transducers [10]. This method involves presenting a pure tone of equal level and phase through both transducers to the user, which gives the sensation of a centrally-located sound source. The user can then adjust the phase or level of one tone to decrease the perceived sound in one ear, leading to a shift in the perceived sound location from the centre to either the left or right side, a process termed as “lateralization.” When lateralization is at its peak, the phase and level data are recorded. The collected data can then be used to create a crosstalk compensation filter, which could potentially serve as a foundation for a crosstalk cancellation system in a living human head [10,11].

### Motivation for the proposed method

Although the “two-BT” technique has shown efficacy in reducing crosstalk within the frequency range of 1 kHz to 4 kHz, it encounters limitations when applied to frequencies below 1 kHz [11]. The sensitivity to phase differences between the cochleae in low-frequency pure tones creates ambiguity in the lateralization cues. This ambiguity complicates the task of phase and level adjustment by participants during sound lateralization tasks [11]. As a result, the utility of the “two-BT” method is essentially confined to frequencies above 1 kHz. Given the crucial role of localization cues at low frequencies [12], the development of a method capable of addressing crosstalk cancellation specifically for frequencies below 1 kHz is of utmost importance.

**Fig. 2:**
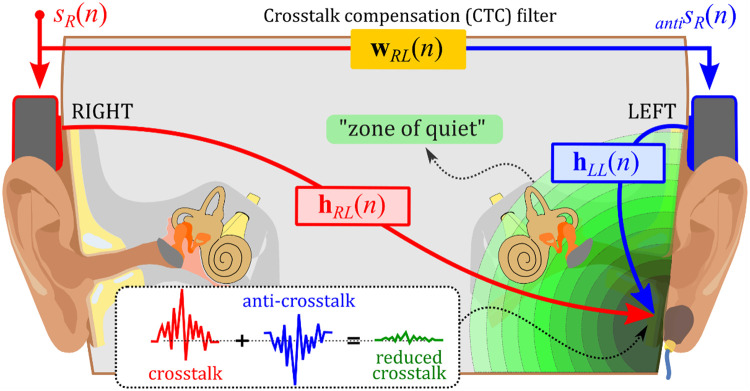
Illustration of unilateral crosstalk cancellation: An accelerometer on the mastoid serves as the target cancellation site. The right bone transducer receives the input signal, while the left one receives the same signal processed through a crosstalk compensation (CTC) filter, creating an anti-signal. These signals cancel each other out at the accelerometer, generating a “zone of quiet” potentially extending to the cochlea in the inner ear.

In this paper, we detail a method for cancelling low-frequency crosstalk that takes advantage of the “zone of quiet” concept from active noise control (ANC) [13]. This method employs an adaptive algorithm to generate the necessary anti-signal for crosstalk cancellation at a strategically placed error sensor, thereby creating a zone of significantly reduced sound around the sensor. The size of this zone is frequency-dependant, with lower frequencies yielding larger zones. When the error sensor is positioned in proximity to the cochlea, such as within the ear canal or on the mastoid, the “zone of quiet” (depicted in Fig. 2) can extend to the cochlea within the inner ear, facilitating the effective cancellation of low-frequency crosstalk. By leveraging the “zone of quiet” concept, this method presents a potential improvement in crosstalk cancellation at low frequencies, where psychoacoustic-based methods like the “two-BT” method encounter limitations.

### Rationale for crosstalk cancellation and its evaluation

Our study is built on the assumption that a reduction in crosstalk sound, whether at the mastoid or in the ear canal, as detected by an accelerometer or a probe microphone, respectively, will correspond to a perceptible change at the cochlea. Using the specific methodology presented in this paper, which is focused on achieving cancellation at the respective sensor locations, we designed a hearing threshold experiment under conditions with and without crosstalk cancellation. The purpose of this experiment is to precisely identify the frequencies at which this reduction is most perceptible at the cochlea.

### Experimental setup

In the method detailed in this paper, crosstalk cancellation is aimed at one ear—the ear on the side of the sensor, as illustrated in Fig. 2. This approach is hence referred to as “unilateral crosstalk cancellation.” The following section elaborates on the implementation specifics of our method, which builds on our previous works [14], [15], [16], [17]. We describe the audio setup and the positioning of bone transducers and sensors on the subject's head. Moreover, a step-by-step guide for constructing the system is provided, along with an analysis of the subjective evaluation method used and a discussion on the limitations of the proposed method.

### Equipment and software

To implement and evaluate our crosstalk cancellation method, we used a combination of specialized equipment and custom software. We have prepared two tables for a comprehensive overview of the resources utilized. Table 1 details the specific equipment used, including bone transducers, sensors, amplifiers, and other hardware. Table 2 provides information about the software and specific packages we utilized for playback, recording, signal processing, and hearing threshold measurement. These tables offer a helpful reference for replicating our experimental setup.

**Table 1:** List of recommended equipment necessary for implementing and evaluating crosstalk cancellation.

Type	Recommended	Used
Bone Transducer (2 units)	Any audiometric bone transducer	Radioear B81 (12.5 ohm)
Elastic Headband (1 unit)	• Radioear's AMBAND BC headband [18] or • Any adjustable elastic headband can be used, but the static force needs to be controlled.	A non-standard elastic headband with a controlled static force within 2.5–3.0 N [19].
Probe Microphone (1 unit)	Any low-noise probe microphone	ER10B+ (Etymotic Research)
Microphone Preamplifier (1 unit)	Probe microphone typically includes a preamplifier	Etymotic Preamplifier
Accelerometer (1 unit)	Any highly sensitive accelerometer	PCB 352A24 – 10.2 mV/(m/s^2^)
Signal Conditioner (1 unit)	Consider input/output range, excitation voltage, etc. when selecting a signal conditioner.	Onosokki PS-1300
Audio Interface (1 unit)	Any high-quality audio interface	Sound Blaster Omni Surround 5.1
Laptop or PC (1 unit)	Any laptop or PC	Lenovo IdeaPad 5 AMD Ryzen 7 5700 U with Ubuntu Studio OS
Monitor and Mouse (1 each)	Any monitor and mouse	Portable monitor with a wireless mouse
Earplugs (1 pair)	High-quality foam earplugs	3 M Foam Earplugs
Acoustic Environment	Audiology-grade soundproof room	Anechoic room

**Table 2:** Overview of software tools and capabilities utilized for audio playback, recording, and signal processing.

Capability	Recommended	Used
Playback and Recording	Any playback and recording software	ALSA with its command-line utilities (e.g., “aplay” and “arecord”)
Signal Processing	Any programming language for signal processing	Python (NumPy, SciPy, and other libraries)
Pure-Tone Hearing Threshold Measurement	Any GUI library compatible with the chosen programming language	IPyWidgets used to create a GUI with 3-button interface for pure-tone selection.

### Audio setup

**Fig. 3:**
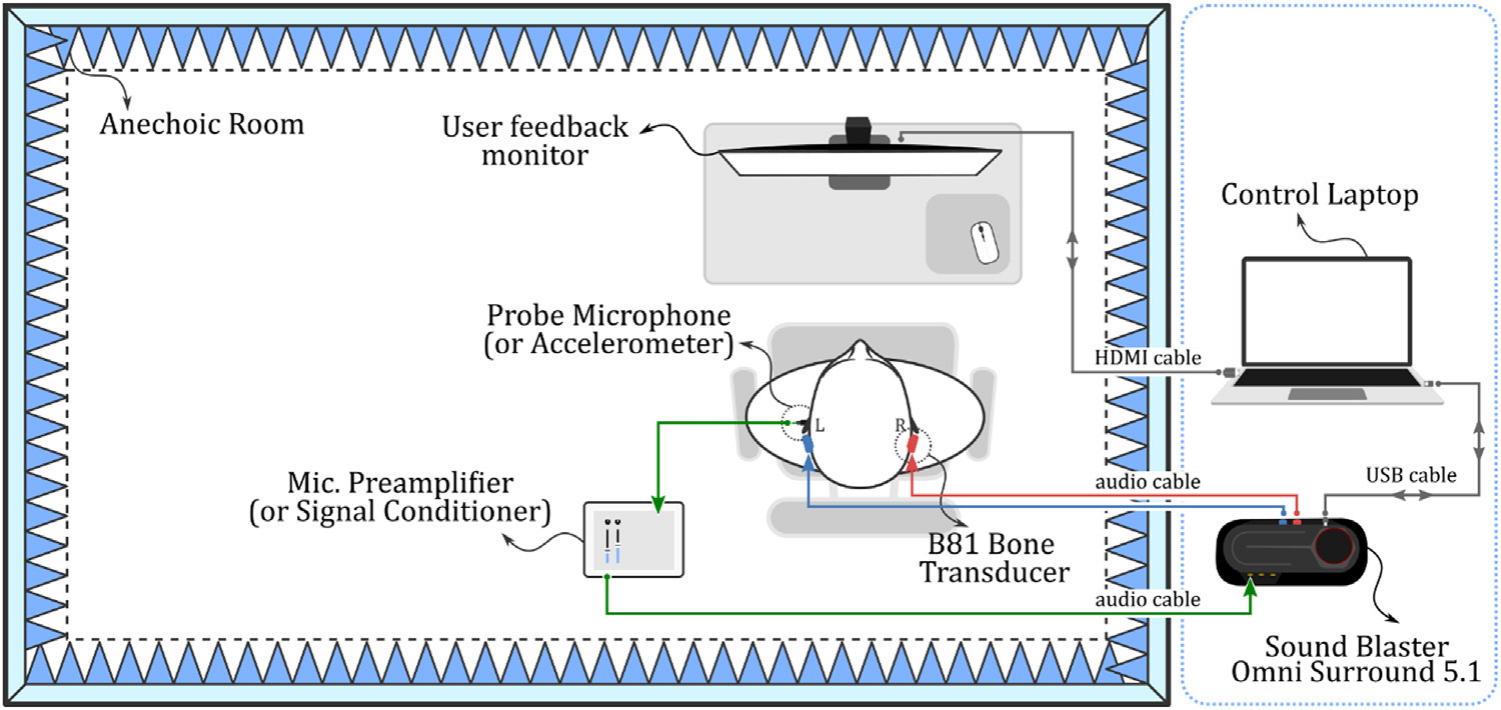
Experimental setup in an anechoic room: placements and connections of sensor, bone transducer, monitor, audio interface, preamplifier, and laptop. The user is seated facing the monitor.

We performed our experiments using the setup illustrated in Fig. 3, which involved a user comfortably seated in front of a monitor with bone transducers and a sensor mounted on their head. The control laptop delivered audio signals to the Sound Blaster Omni Surround 5.1 audio interface through a USB 2.0 cable. These signals were then relayed to the bone transducers via RCA connector audio cables, and as they traversed the user's head, the sensor recorded them. Either a probe microphone or an accelerometer could serve as the sensor, and it was linked to an amplifier or a signal conditioner to boost its faint signals. The amplified signals were subsequently dispatched to the audio interface through a TRS jack connector. Custom Python scripts operating on the laptop controlled the experiments, while the monitor and mouse facilitated the collection of user responses during hearing threshold measurements. This setup enabled us to acquire the necessary data to assess the efficacy of our unilateral crosstalk cancellation method.

### Bone transducer placement

**Fig. 4:**
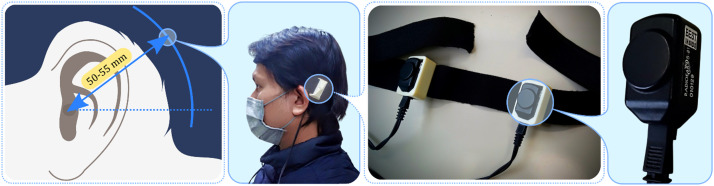
Detailed illustration of the placement of B81 bone transducers on the human head. The transducers are positioned specifically 50 to 55 mm behind the ear and slightly above the ear canal on the temporal bone, and are attached to a headband using custom-housing cases. The orientations of the transducers, at either the 10 o'clock or 2 o'clock position depending on the side of the head, are also indicated.

The placement of bone transducers followed a configuration analogous to a bone-anchored hearing aid (BAHA) setup. Two bone transducers, secured to an elastic headband with 3D-printed custom housing cases for a comfortable fit, were positioned on the user's head at about 50 to 55 mm behind the ear and slightly above the ear canal on the temporal bone. This location is known to be optimal for transmitting sound through bone conduction [20]. Depending on whether they were on the left or right side of the head, respectively, the transducers were situated at the 10 o'clock or 2 o'clock position. If a non-standard headband was chosen, we adjusted it to exert a static force of 2.5 to 3.0 N, in accordance with Reinfeldt et al.’s recommendations, to secure effective acoustic coupling [19]. Although the AMBAND headband, providing a coupling force of approximately 5.0 N within the audiometer standards’ range [18], could serve as an alternative, we opted not to use it due to the discomfort it caused at the contact point with the transducer, especially considering the length of our experiments (exceeding one and a half hours). The exact locations of the transducers on the user's head are illustrated in Fig. 4.

### Sensor placement

**Fig. 5:**
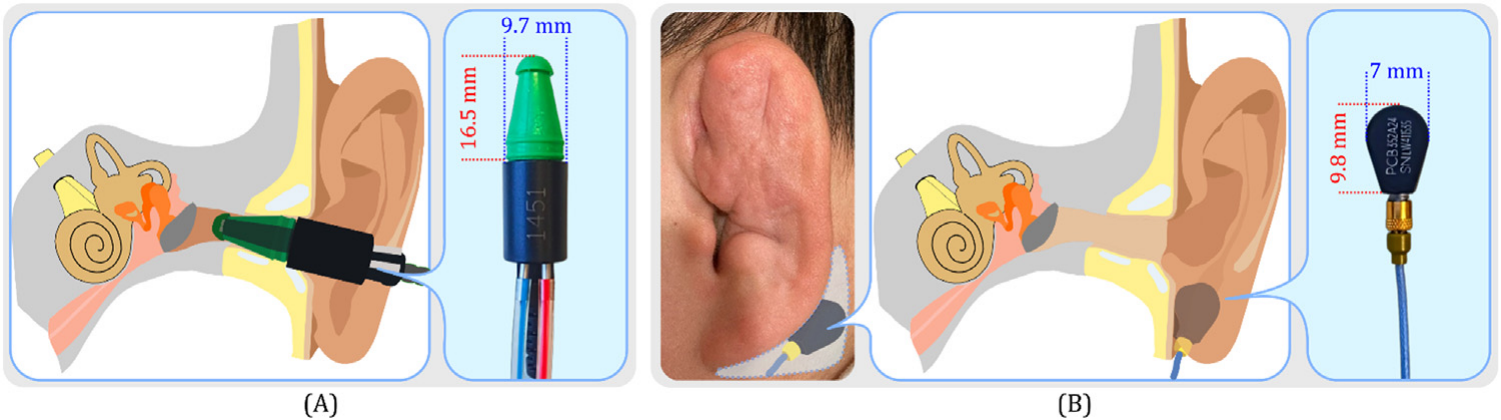
Visual representation of sensor placement. Panel (a) displays the location and dimensions of the ER10B+ probe microphone inserted into the left ear canal. Panel (b) illustrates the alternate use of an accelerometer, including its location and dimensions on the mastoid, employed when microphone insertion is unfeasible, such as in cases of auricular haematoma.

The placement of the sensor is essential for achieving optimal crosstalk cancellation at the cochlea. In our study, we focused on two potential cancellation sites: the ear canal and the mastoid. We primarily targeted the ear canal, as research has shown that the ear canal sound pressure (ECSP) is subject to change as bone-conducted sounds propagate through the human head [21]. Furthermore, a strong correlation between ECSP and hearing thresholds at most tested frequencies has been reported by Reinfeldt et al. [21], suggesting that crosstalk cancellation in the ear canal could potentially extend to the cochlea in the inner ear. We utilized an ER10B+ probe microphone from Etymotic, positioned in the left ear canal, to measure ECSP as an indicator of successful cancellation. However, certain conditions such as aural atresia [22] or auricular haematoma might render the ear canal inaccessible. In these instances, we deployed a highly sensitive accelerometer, the PCB 352A24, on the mastoid as an alternative approach to detect bone-conducted sounds. Fig. 5 offers a visual representation of the precise locations on the user's head where the probe microphone and accelerometer were situated for our experiments.

### Transducer equalization

**Fig. 6:**
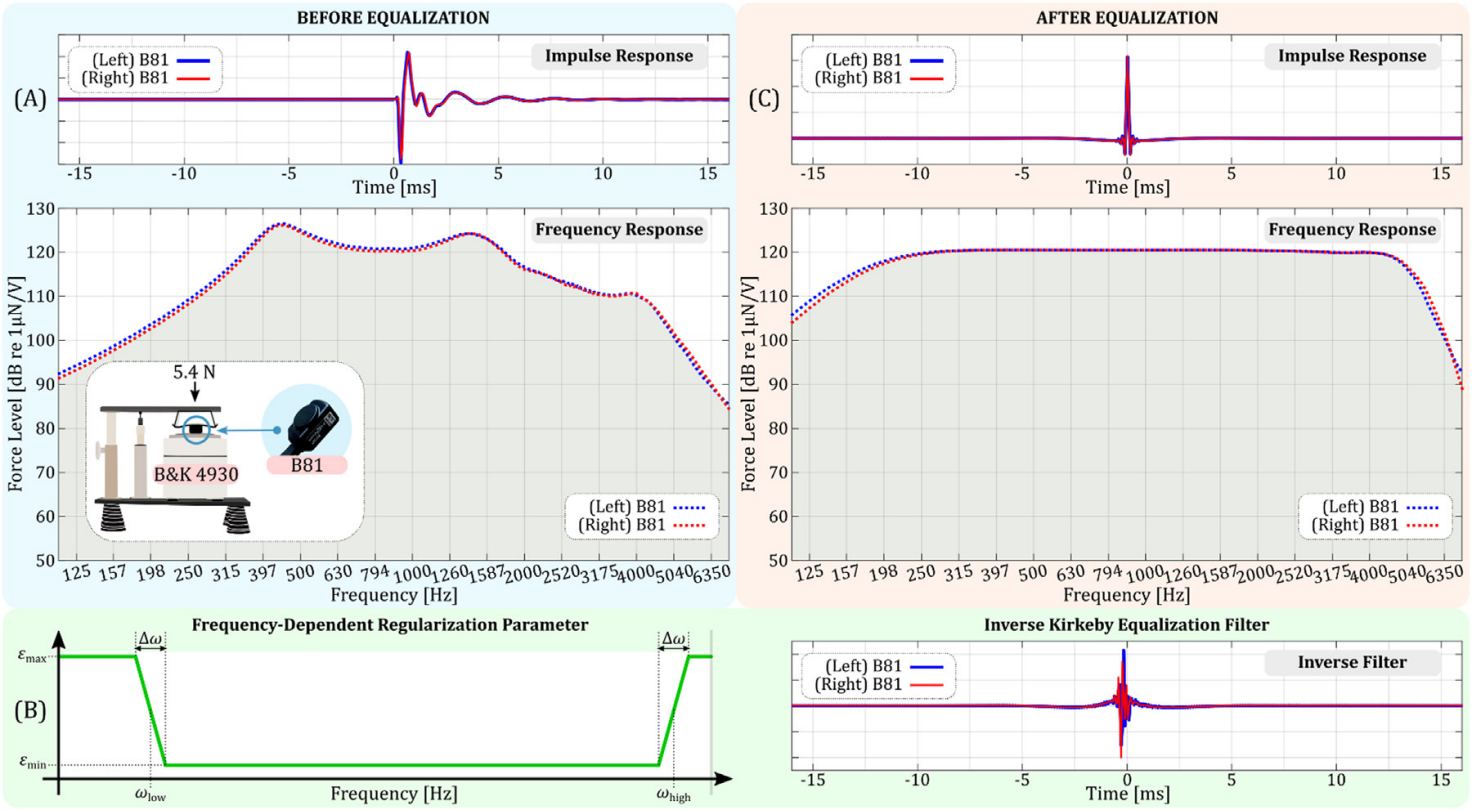
Equalization results for B81 bone transducers. (a) Shows the original characteristics of the transducers, with the top panel depicting impulse responses and the bottom panel displaying their frequency responses; an inset within the bottom panel details the placement of the B81 bone transducer on the B&K 4930 artificial mastoid. (b) Demonstrates the equalization methodologies, with the left panel illustrating the frequency-dependant regularization function applied in the Kirkeby algorithm and the right panel presenting the inverse Kirkeby equalization filters derived from the B81 transducers’ impulse responses. (c) Presents results post-equalization, where the top panel reveals the equalized impulse responses, and the bottom panel emphasizes the achieved flat frequency response within the desired range.
❖ *Step 2 – Frequency Response Measurement*: We played the time-stretched pulse (TSP) signal [24] through the transducer on the artificial mastoid. The mastoid's output was amplified with the B&K 2690 amplifier and converted to a force level. By deconvolving this output with the inverse TSPs, we derived the impulse response (top panel) and its frequency response (bottom panel), as illustrated in Fig. 6(a). A comparison between the left and right transducers showed a minimal difference of under 1.5 dB in their frequency responses, indicating their near-identical behaviour.
❖ *Step 3 – Application of the Kirkeby Algorithm*: We represent the left and right B81 impulse responses as $t_{L}\left(n\right)$ and $t_{R}\left(n\right)$. First, we transformed these responses to the frequency domain using FFT, expressed as $T_{i}\left(\omega \right)=\text{FFT}\left\{t_{i}\left(n\right)\right\}$, where i stands for either L (left) or R (right). Next, in the frequency domain, we computed the inverse filter:
  $$C_{i}\left(\omega \right)=\;\frac{\text{conj}\left\{T_{i}\left(\omega \right)\right\}}{\text{conj}\left\{T_{i}\left(\omega \right)\right\}\cdot T_{i}\left(\omega \right)+\varepsilon \left(\omega \right)}$$
  The regularization parameter, ε(ω), which is frequency-dependant, limits the equalization range. This parameter is illustrated in Fig. 6(b)’s left panel, and we adjusted it manually for the desired response. A minimal $\varepsilon_{min}$ value was set within the $\omega_{low}$ to $\omega_{high}$ frequency range. Using the IFFT, we reverted the inverse filters to the time domain, represented by $c_{i}\left(n\right)=\text{IFFT}\left\{C_{i}\left(\omega \right)\right\}\;$for i=L,R. The results are displayed in the right panel of Fig. 6(b).
❖ *Step 4 – Applying the Inverse Kirkeby Equalization Filters*: We convolved the inverse Kirkeby filters (shown in Fig. 6(b)) with the measured impulse responses from Fig. 6(a) to obtain equalized impulse responses. The results, displayed in Fig. 6(c), clearly show that the equalized frequency response closely matches a "flat" curve in our desired frequency range.

For our crosstalk cancellation method, measuring the impulse responses between transducers and sensor is essential. Yet, transducers often have inherent imperfections in their frequency responses, making them not flat. Equalization aims to rectify this, producing a flat or desired frequency response. At frequencies where the transducer has a weak response, equalizing can lead to large filter coefficients or “boosts.” If these boosts are too extreme, they can damage the transducer. To manage this, we use the Kirkeby algorithm [25], which introduces regularization parameters. These parameters control the derived inverse filter, preventing excessive amplification of certain frequencies. For our work, we concentrated on equalizing frequencies between 200 and 4000 Hz. The steps for this process are detailed below:

❖ *Step 1 – Measurement Setup Based on B81 Datasheet*: Following the B81 datasheet [23], we used the B&K 4930 artificial mastoid for calibration. This device has an adjustable loading-arm that allows for static forces between 2 N and 8 N. As recommended by the datasheet, we set the B81 on the calibration surface with a force of 5.4 N, as illustrated in Fig. 6(a).

In the next section, the derived inverse Kirkeby equalization filters will be used to convolve with the impulse responses $h_{RL}\left(n\right)$ and $h_{LL}\left(n\right)$ measured within the human head, minimizing the influence of the transducer.

### Step-by-step method

The implementation of a unilateral crosstalk cancellation system for bone conduction requires a sequence of steps. For clarity and comprehensibility, these steps have been divided into three subsections: (1) *Estimation of impulse responses*; (2) *Estimation of crosstalk compensation (CTC) filter*; and (3) *Verification of the effectiveness of the CTC filter*. Each subsection provides detailed descriptions of its corresponding step. In addition to the text, a video has been included to provide a visual demonstration of the system in operation.

### Estimation of impulse responses

**Fig. 7:**
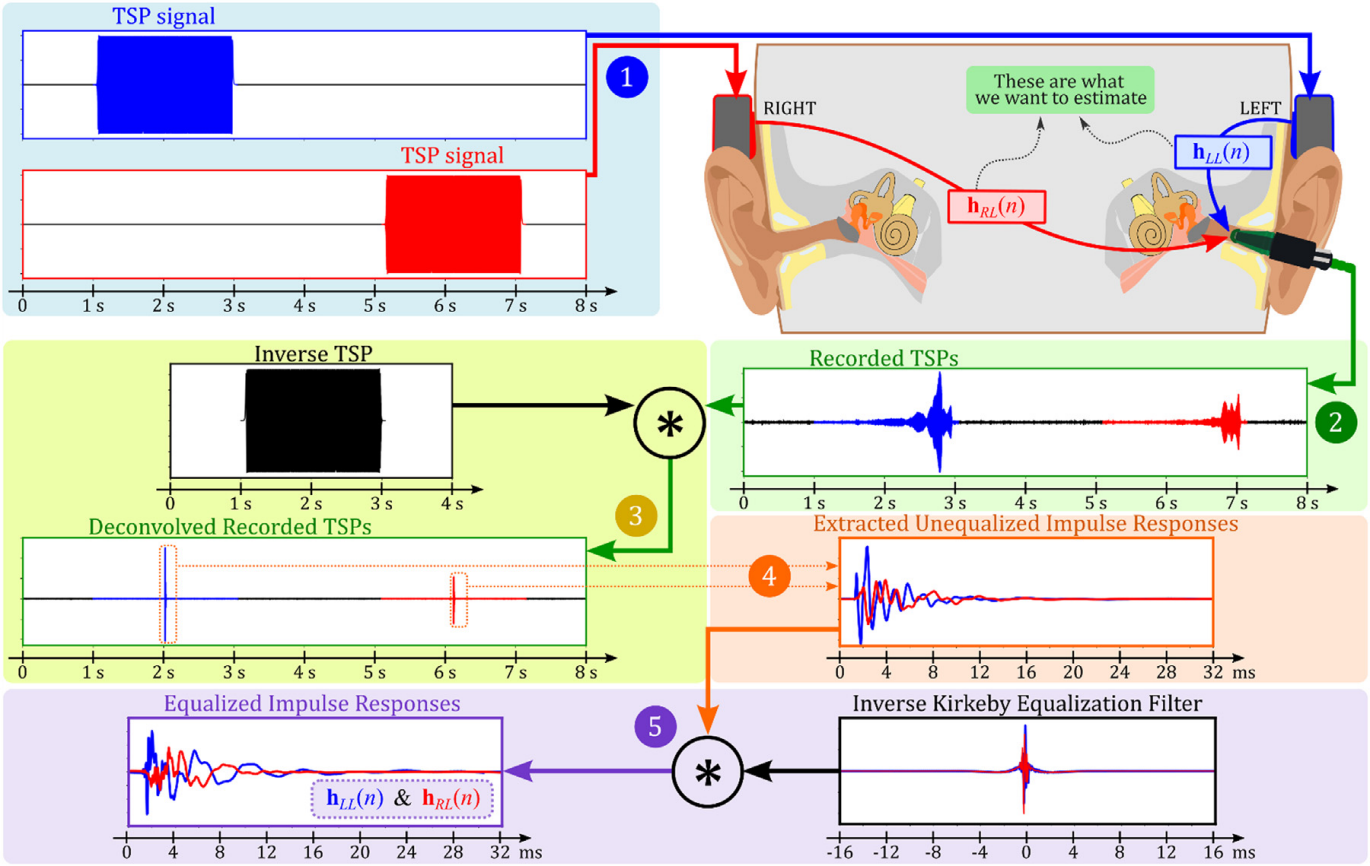
Illustration of the five-step process for acquiring impulse responses $h_{RL}\left(n\right)$ and $h_{LL}\left(n\right)$: (1) Generation of a stereo signal with time-stretched pulses (TSPs) in both channels, staggered by a certain delay; (2) Transmission of the stereo signal through bone transducers and recording of the resultant response using a probe microphone; (3) Deconvolution of the recorded TSP signal with its inverse; (4) Extraction of 32 ms unequalized impulse responses from the deconvolved TSPs; and (5) Convolution of the unequalized impulse responses with the inverse Kirkeby equalization filters to obtain $h_{RL}\left(n\right)$ and $h_{LL}\left(n\right)$ where the transducer effect has been minimized.

This subsection's primary objective is to estimate the impulse responses, $h_{RL}\left(n\right)$ and $h_{LL}\left(n\right)$, between the right and left transducers and the error sensor, which can either be an ER10B+ probe microphone or an accelerometer. When using the ER10B+ probe microphone, it is essential to acknowledge the occlusion effect. This effect arises when the ear canal is blocked, leading to a noticeable boost in low-frequency components. In our experiments, we intentionally measured impulse responses that inherently included this effect, choosing to integrate it as a characteristic of the response rather than treating it as an unwanted artefact that might need to be removed. Given the ER10B+’s design, primarily to capture internal ear canal sounds, our impulse responses are predominantly influenced by the sound within the ear canal, with minimal interference from external sources. Conversely, the accelerometer, positioned on the mastoid, does not present concerns related to the occlusion effect. These impulse response estimations are pivotal in determining the crosstalk compensation (CTC) filter using adaptive algorithms, such as the filtered-x least mean squares (FxLMS) algorithm. This algorithm seeks to cancel crosstalk sound from the right bone transducer by employing an anti-sound from the left transducer (as referenced in Fig. 2). The process of acquiring impulse responses, encompassing five steps, is illustrated in Fig. 7. For our measurements, we utilized the time-stretched pulse (TSP) signal, or swept sine, as proposed by Suzuki *et al*. [24].

❖ Step 1 – Signal Preparation: We generated a stereo signal, each audio channel of which contained a TSP signal, separated by the length of the TSP signal (32,728 samples or 2.05 s at a 16 kHz sampling rate). The TSP signals in the left and right channels began at 1 s and 5.1 s, respectively, with the output voltages from the audio interface set to −20 dB re 1VRMS (see Step 1 of Fig. 7).
❖ Step 2 – Signal Presentation and Recording: Using bone transducers, we delivered the stereo signal to the user's head and, depending on which sensor was in use, either recorded the change in ECSP induced by bone-conducted sounds in the left ear canal with a probe microphone or measured the acceleration level at the left mastoid using an accelerometer. After applying a high-pass filter with a 100 Hz cutoff frequency, we successfully observed the two TSP signals (refer to Step 2 of Fig. 7).
❖ Step 3 – Deconvolution: We deconvolved the recorded TSP signal with its inverse TSP to obtain two impulse responses (see Step 3 of Fig. 7).
❖ Step 4 – Unequalized Impulse Response Extraction: Despite the TSP signal being 32,728 samples in length, we extracted unequalized impulse responses of just 512 samples (32 ms) in length. Given the closeness of the transducer and sensor on the user's head, this duration was deemed sufficient (refer to Step 4 of Fig. 7).
❖ Step 5 – Equalization: To minimize the transducer effect, we convolved the extracted unequalized impulse responses (refer to Step 5 of Fig. 7) with the inverse Kirkeby equalization filters, as described in detail in the “Transducer Equalization” section. The equalized impulse responses $h_{RL}\left(n\right)$ and $h_{LL}\left(n\right)$ were then used to estimate the CTC filter, which is elaborated on in the next subsection.

Note that the choice of signal for measuring impulse responses may depend on whether the transducer exhibits nonlinear behaviour. In our experiments, the TSP signal was appropriate given its compatibility with the B81 audiometric bone transducer, which is characterized by its low harmonic distortions [26]. Conversely, when using the B71 transducer, which is known for its pronounced harmonic distortions [26], exponential sweeps [25,27] could be a more fitting choice to account for these distortions.

### Estimation of crosstalk compensation (CTC) filter

The design and estimation of the CTC filter play a crucial role in achieving effective crosstalk cancellation. While there are many methods available for determining the CTC filter, this work specifically uses the Filtered-x Least Mean Squares (FxLMS) algorithm. We chose this due to the algorithm's simplicity and the proven effectiveness of the FxLMS method for our specific application. It is worth noting that the FxLMS is not the only available approach. For instance, the closed-form solution proposed in [28] offers an alternative method for CTC filter design. The method emphasizes frequency-dependant regularization, which allows the optimization of FIR filters within specific frequency bands. Such an approach aligns with our objective of limiting the CTC filter to cancel low-frequency crosstalk. However, in this research, we focus on the FxLMS approach to ensure consistency with our conducted experiments.

Following the overview of CTC filter estimation methods, it is essential to delve deeper into the specifics of our approach. The primary path $h_{RL}\left(n\right)$ refers to the crosstalk sound's route from one bone transducer (e.g., the right one) to the opposite ear. In contrast, the secondary path $h_{LL}\left(n\right)$ represents the route for the anti-crosstalk sound emitted by the opposing transducer (e.g., the left one). We estimated these impulse responses using TSP signals in the previous subsection. The steps for estimating the CTC filter, conducted through computer simulation, used these previously measured impulse responses $h_{RL}\left(n\right)$ and $h_{LL}\left(n\right)$:

❖ Step 1 – Initialize Variables: We set the CTC filter $w_{RL}\left(n\right)$ length (p) to 512 samples and initialized the filter coefficients $w_{RL}^{T}\left(n\right)=\left[w_{RL_{0}}\left(n\right),w_{RL_{1}}\left(n\right),\ldots ,w_{RL_{p-1}}\left(n\right)\right]$ to zero.
❖ Step 2 – Acquire Input Signals: We utilized white noise filtered by a 224–1122 Hz bandpass as the input signal $s_{R}\left(n\right)$, targeting a 250–1000 Hz cancellation frequency range. We collected a block of input signal $s_{R}\left(n\right)$ of length p: $s_{R}\left(n\right)={\left[s_{R}\left(n\right),\;s_{R}\left(n-1\right),\;\ldots ,s_{R}\left(n-p+1\right)\right]}^{T}$.
❖ Step 3 – Filtered-x Signal: The block input signal $s_{R}\left(n\right)$ was convolved with the secondary path impulse response estimate $c_{LL}\left(n\right)$ to obtain the filtered-x signals $s_{Rf}\left(n\right)=s_{R}^{T}\left(n\right)\;c_{LL}\left(n\right)$. Note that $c_{LL}\left(n\right)$ is the same as $h_{LL}\left(n\right)$ in our simulation.
❖ Step 4 – Update CTC filter coefficients: For each time step n within the block of length p, the filter coefficients $w_{RL}\left(n\right)$ were updated using the least mean squares (LMS) algorithm. This involved:

(a) Calculating the output of the CTC filter${\;}_{anti}s_{R}\left(n\right)$ at time step n:$\;{\;}_{anti}s_{R}\left(n\right)=s_{R}^{T}\left(n\right)\;w_{RL}\left(n\right)$.
(b) Calculating the error signal e(n) at time step n: $e\left(n\right)=s_{R}^{T}\left(n\right)\;h_{RL}\left(n\right)+{\;}_{anti}s_{R}^{T}\left(n\right)\;h_{LL}\left(n\right)$.
(c) Updating filter coefficients: $w_{RL}\left(n+1\right)=w_{RL}\left(n\right)-\mu \;e\left(n\right)\;s_{Rf}\left(n\right)$ where μ denotes the learning rate, which controls the convergence speed and stability of the algorithm.

**Fig. 8:**
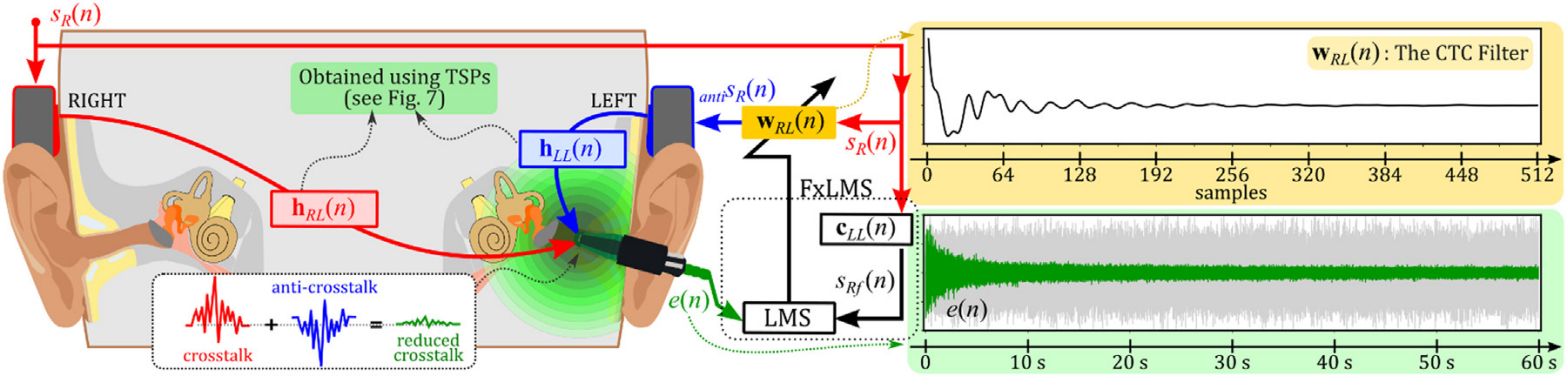
Application of the FxLMS algorithm for crosstalk cancellation in bone conduction. The left panel presents a block diagram illustrating the CTC filter estimation process, which aims to cancel the crosstalk sound from the right bone transducer (red) by using the anti-crosstalk sound from the left transducer (blue). The impulse responses of the primary and secondary paths (indicated by arrows) were obtained using TSP signals (as shown in Fig. 7), and a computer simulation of the FxLMS algorithm followed. The top right panel displays the final CTC filter after convergence of the error signal, while the bottom right panel demonstrates the convergence of the error signal (green). A light-grey curve is also presented for comparison, representing the error signal without the CTC filter.

❖ *Step 5 – Iterate*: We repeated steps 2–4 for a predefined number of iterations (60 s in our case). The change in the CTC filter coefficients becomes negligible at this point (see top right panel of Fig. 8). The final coefficients represent the CTC filter that minimizes the crosstalk sound at the sensor location.

This process is key for successful crosstalk cancellation in bone conduction applications, as it effectively estimates the CTC filter using the FxLMS algorithm.

### Verification of the effectiveness of the CTC filter

**Fig. 9:**
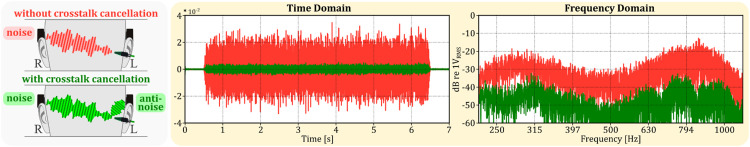
Reduction of crosstalk sound in the left ear canal through the use of the CTC filter, as determined by simulation. The left panel illustrates a user's head in two different scenarios: red, which represents the condition without crosstalk cancellation, and green, which represents the condition with crosstalk cancellation. The right panel demonstrates the recorded crosstalk sound in the left ear canal with both time-domain (left plot) and frequency-domain (right plot) representations. The colour green denotes the crosstalk sound with cancellation, indicating a successful reduction.
❖ Step 3 – Data Analysis: We compared the recorded crosstalk sounds in both the time and frequency domains. A decrease in level when the anti-noise signal was added confirmed the effectiveness of the CTC filter in minimizing crosstalk. The comparison plots can be found in the right panel of Fig. 9.
❖ Step 4 – Reestimation of Impulse Responses and CTC Filter (if necessary): If the crosstalk sound was not successfully reduced or cancelled, it may have been due to the transducers shifting from their original positions. In this case, we reestimated the impulse responses and recalculated the CTC filter. We then repeated Steps 1–3 to verify the effectiveness of the updated CTC filter.

In this subsection, our objective is to validate the effectiveness of the CTC filter, which we obtained via computer simulation in the preceding subsection. To perform this verification experiment, we adhered to the following steps:

❖ Step 1 - Signal Preparation: We created a 6-second white noise signal filtered by a bandpass filter, the cutoff frequencies of which ranged from 224 to 1122 Hz.
❖ Step 2 - Signal Presentation and Recording: We played the noise signal through the right transducer and the anti-noise signal, generated using the CTC filter, through the left transducer. This was done with the same subject from whom the impulse responses $h_{RL}\left(n\right)$ and $h_{LL}\left(n\right)$ were initially obtained. The left panel of Fig. 9 provides a visual representation of this setup. We recorded the crosstalk sounds at the sensor location both with and without the addition of the anti-noise signal.

The successful implementation of a crosstalk cancellation system in bone conduction applications relies heavily on verifying the effectiveness of the CTC filter. The results of our verification process, which showcase a successful reduction in crosstalk sounds, are depicted in the right panel of Fig. 9. To further illustrate the process, we developed a real-time crosstalk cancellation demonstration using a white noise (224–1122 Hz) input signal and a simple graphical user interface (GUI) to control the presentation of the anti-noise from the left transducer [16]. A video of our demonstration can be found here: https://youtu.be/D-Oy1AyythQ.

### Subjective evaluation

The primary aim of this section is to ascertain if the crosstalk cancellation, specifically designed to take place at the sensor location, indeed extends effectively to the cochlea in the inner ear. We do this by employing pure-tone hearing thresholds, measured in noise conditions both with and without the implementation of crosstalk cancellation. The specifics of this evaluation are distributed across three subsections: (1) *Setup*, in which we detail the experimental configuration; (2) *Stimuli*, in which we specify the specific signals used; and (3) *Procedure*, during which we narrate the systematic steps executed for hearing thresholds measurements.

### Setup

**Fig. 10:**
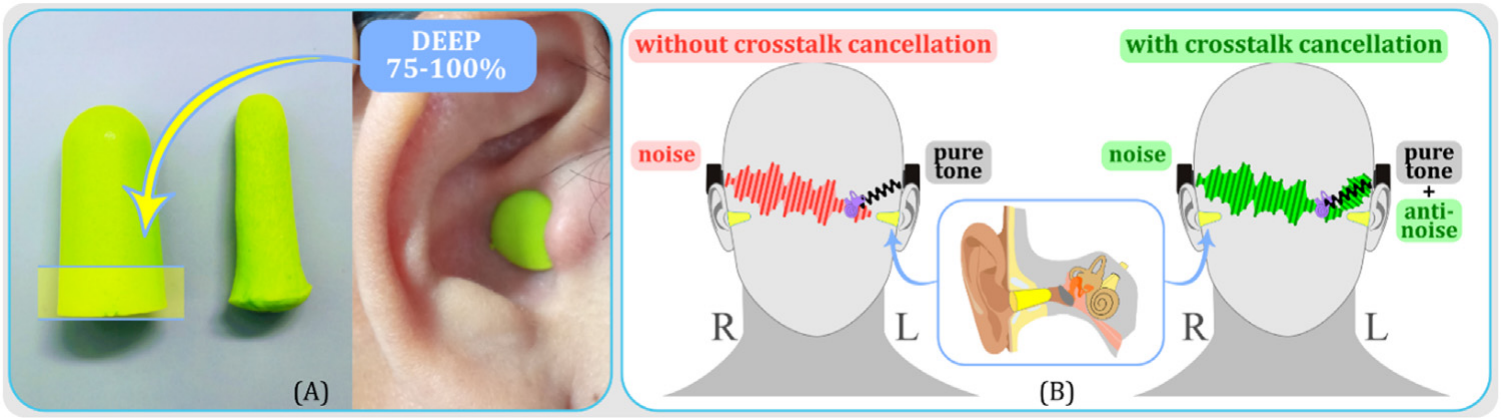
(a) A photograph of an ear with a deeply inserted earplug, simulating bilateral conductive hearing loss. Earplugs were utilized after the successful crosstalk cancellation was confirmed at the sensor location. (b) Illustrations of heads wearing bone transducers and earplugs under two different noise conditions: left illustration displays the setup without crosstalk cancellation (noise on the right transducer, pure-tone on the left), while the right illustration showcases the setup with crosstalk cancellation (noise on the right transducer, pure-tone and anti-noise on the left). Measurements of pure-tone hearing thresholds were taken under both conditions to validate the effective reach of crosstalk cancellation to the cochlea.

The setup for subjective evaluation closely follows the configuration described in the “Experimental Setup” section, but with some distinct differences. After confirming crosstalk cancellation at the sensor location, subjects were instructed to insert earplugs deeply into both ear canals, ensuring that about 75–100 % of the earplug was inside the ear canal, as depicted in Fig. 10(a). This deep insertion, informed by [29], aimed to reduce the volume inside the ear canal, minimizing the occlusion effect. These earplugs served two main functions: first, to prevent any air-conducted sounds originating from the bone transducers from entering the ear canals; and second, to simulate conditions of bilateral conductive hearing loss. Ensuring that the earplugs’ insertion did not alter the positions of the bone transducers was crucial, as any change could potentially disrupt the established cancellation. During the pure-tone hearing threshold measurement (subjective evaluation), we did not use the sensor (either a probe microphone or accelerometer) unless the bone transducers’ positions changed, necessitating a reestimation of the CTC filter.

### Stimuli

This subsection outlines the specific stimuli utilized to measure hearing thresholds under noisy conditions, both with and without crosstalk cancellation. As depicted in Fig. 10(b), the configuration without crosstalk cancellation fed noise into the right transducer and a pure tone into the left one. On the other hand, the setup with crosstalk cancellation introduced noise into the right transducer and a blend of pure tone and anti-noise (calculated using the CTC filter) into the left transducer. We examined pure-tone frequencies of 250, 315, 397, 500, 630, 794, and 1000 Hz. The noise employed was a one-third-octave band noise centred at the frequency under examination. The noise and pure tone had durations of 800 ms and 500 ms, respectively, with the pure tone being temporally centred within the noise. Both the noise and the pure tone had 75 ms fade-in and fade-out times. The participants' task was to discern the pure tone amidst the masking noise, consistently presented at a 30 dB sensation level. The subsequent “Procedure” subsection provides a more detailed explanation of the hearing threshold task using these stimuli.

### Procedure

**Fig. 11:**
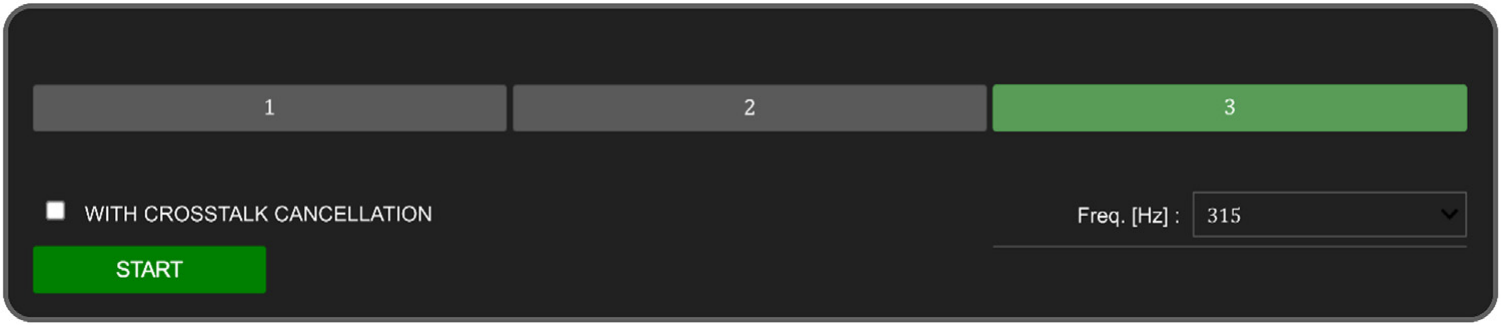
Screenshot of the custom-made graphical user interface (GUI) for executing the 2-down/1-up adaptive three-interval forced-choice (3IFC) procedure. The GUI, developed using Python's IPyWidgets package, features three buttons representing the audio intervals. Each button turns green when the corresponding audio interval is being played.

**Fig. 12:**
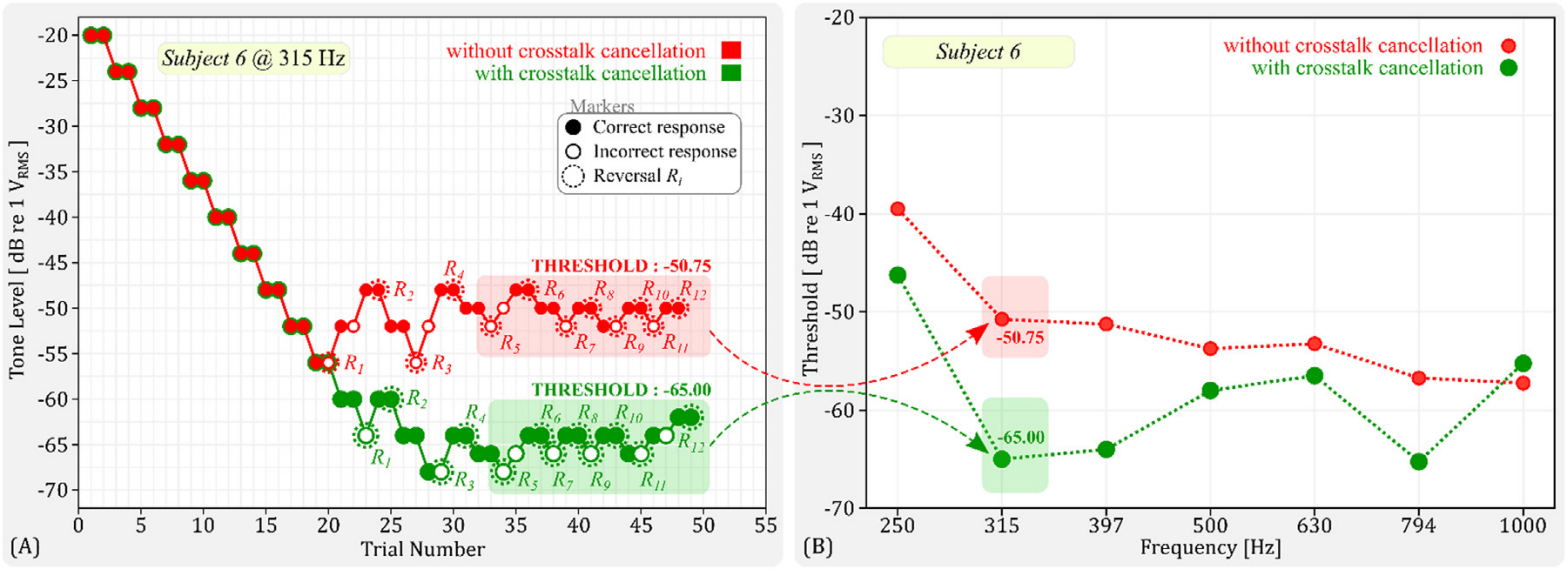
(a) Example outcome of the 2-down/1-up adaptive 3IFC procedure for Subject 6 at 315 Hz. The figure compares the pure-tone hearing threshold measured with crosstalk cancellation (−65 dB re 1V_RMS_) against the threshold measured without crosstalk cancellation (−50.75 dB), highlighting the improvement in hearing thresholds brought about by crosstalk cancellation. (b) Hearing threshold results for the same subject at other tested frequencies (250, 397, 500, 630, 794, and 1000 Hz), further emphasizing the influence of crosstalk cancellation on hearing thresholds across a range of frequencies.

We estimated the hearing thresholds using a 2−down/1−up adaptive three-interval forced-choice (3IFC) procedure. To facilitate this, we developed a custom-made graphical user interface (GUI) using Python's IPy-Widgets package. The GUI, shown in Fig. 11, featured three buttons, each corresponding to an audio interval. The procedure began with the selection of the test frequency and the condition (with or without crosstalk cancellation). The initial intensity level of the pure tone was set at −20 dB re 1V_RMS_. The subject was then presented with three audio intervals, each initiated by a button on the GUI. The button turned green when the corresponding audio was played. The pure tone was randomly assigned to one of the intervals, with noise present in all three. The subject was instructed to listen to all three intervals and identify the one containing the pure tone by clicking the corresponding button. The intensity level was decreased by the step size (4 dB) if the subject correctly identified the interval containing the pure tone in two consecutive trials (2−down). If they failed to do so (1−up), the intensity level was increased by the same step size. After four reversals, the step size was reduced to 2 dB. The procedure continued until a total of 12 reversals were reached, after which we calculated the threshold estimate by averaging the intensity values at the last eight reversals. This process was repeated for all frequencies tested. Fig. 12 shows an example outcome of the 2−down/1−up adaptive 3IFC procedure. In this specific case, the pure-tone hearing threshold measured with crosstalk cancellation at 315 Hz (Fig. 12(a)) was found to be −65 dB re 1V_RMS_. This value is lower than the −50.75 dB threshold measured without crosstalk cancellation, suggesting that crosstalk cancellation led to an improvement in hearing thresholds for this individual. Additionally, Fig. 12(b) shows the hearing threshold results for the same subject at other tested frequencies (250, 397, 500, 630, 794, and 1000 Hz), further demonstrating the impact of crosstalk cancellation on hearing thresholds across various frequencies.

### Method validation

In this section, we put our crosstalk cancellation method to the test. We aim to confirm whether the cancellation detected at the sensor location effectively reaches the cochlea in the inner ear. This validation process unfolds in two parts: (1) *Participants and Supplementary Methodology*: We detail the normal-hearing individuals who took part in our primary study and describe the overall experimental setup. Additionally, to provide context for the earplug insertion depth used in our main experiment, we introduce a supplementary experiment investigating the impact of different earplug insertion depths on the occlusion effect. (2) *Experiment Results and Discussion*: Here, we present and interpret the findings from the subjective evaluation. This evaluation not only includes results from the primary crosstalk cancellation method but also integrates data from the supplementary occlusion effect experiment. We measure pure-tone hearing thresholds under noise conditions with and without crosstalk cancellation and discuss data concerning the reduction of crosstalk at the sensor location.

### Participants and supplementary methodology

*Participants*: Seven individuals, five males and two females aged between 21 and 32 years, participated in this study. Each of them had normal hearing, confirmed via pure-tone audiometry, with hearing thresholds all below 20 dB HL. Prior to the experiment, an otoscopic examination was conducted to ensure that no excessive earwax was present, as it could potentially pose a risk when inserting earplugs deeply. All participants provided their written informed consent before the experiment commenced. Participants wore a pair of audiometric bone transducers, as depicted in Fig. 4, and described in the “Experimental setup” section. The targeted cancellation sites were the left ear canal, using a probe microphone (Fig. 5(a)), and the left mastoid, using an accelerometer (Fig. 5(b)). Due to the scope of our evaluation, which included two sensor locations, seven low frequencies, and two conditions (with and without crosstalk cancellation), the experiment took over an hour and a half to complete for a single location or sensor. To accommodate this, the experiment was conducted across two separate days, scheduled according to each participant's availability.

*Supplementary Methodology*: Due to the potential occlusion effect from our main study's foam earplugs—an increase in sound perception through BC [29]—we conducted a supplementary test focusing on earplug insertion depths: Shallow (∼6 mm), Mid-depth (∼12 mm), and Deep (18–24 mm). The occlusion effect was quantified by comparing BC hearing thresholds with and without earplug occlusion. Audiometric tests were conducted using the Resonance's R17A-BC audiometer and the same bone transducers depicted in Fig. 4 from our primary experiment. For efficiency, measurements were limited to participants’ left ears, with the non-test ear masked via a Radioear DD45 audiometric headset using narrow band noise (−24 dB/oct slope) centred on the test frequency. Thresholds were measured at octave intervals from 250 to 4000 Hz, starting at 1000 Hz. We began with a clearly audible tone (around 30 dB or higher). The intensity was then decreased in 10 dB increments until it became inaudible to participants. When undetected, the intensity was increased in 5 dB steps until it was perceived again. This iterative process, known as “bracketing,” involved alternately decreasing and increasing the tone. The lowest intensity where the tone was detected in at least two out of three presentations was recorded as the threshold. After measuring non-occluded thresholds, we proceeded with testing across different earplug depths, maintaining consistent bone transducer positioning. Completing the supplementary experiment took approximately 20–30 min per participant across all conditions.

### Experiment results and discussion

**Fig. 13:**
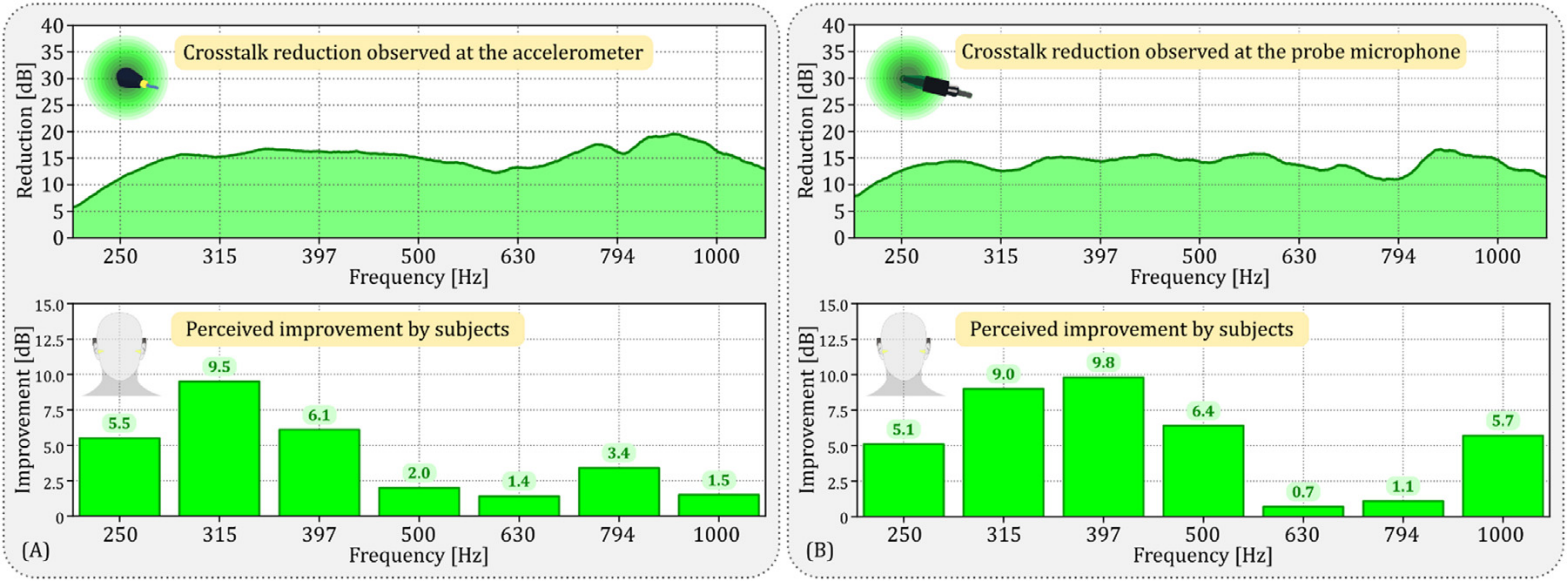
Crosstalk cancellation results at two target locations: (a) mastoid (using an accelerometer) and (b) ear canal (using a probe microphone). Each subfigure includes two panels: the top panel shows average crosstalk reduction at the target location across seven subjects; the bottom panel presents average hearing threshold improvements from subjective evaluations (hearing threshold measurements) for the same seven subjects. Note: Data in Fig. 12(a) was previously published in [17].

Our experiment demonstrated substantial crosstalk reduction at both target cancellation locations: the ear canal and the mastoid. As evidenced in the top panel of Figs. 13(a) and (b), we achieved a crosstalk reduction exceeding 10 dB across seven subjects within the frequency range of 250 to 1000 Hz. The average crosstalk reduction across frequencies was 15.9 dB for the accelerometer on the mastoid, and 14.2 dB for the probe microphone in the ear canal.

In our hearing threshold measurements, both with and without the anti-noise signal, we found that the hearing threshold improvements, as shown in the bottom panel of Figs. 13(a) and (b), were not as significant as the crosstalk reductions recorded by the sensors. These improvements were derived from the differences between the mean hearing thresholds without and with crosstalk cancellation, as listed in Table 3. The average improvements across subjects and frequencies were 4.2 dB and 5.4 dB when the anti-signal was estimated by the CTC filter using the accelerometer and probe microphone, respectively. Notably, we observed significant improvements at certain specific frequencies. When considering the accelerometer, the most significant improvements were at 315 Hz (9.5 dB, *p* = 0.00018), with notable enhancements also at 250 Hz (5.5 dB, *p* = 0.03575) and 397 Hz (6.1 dB, *p* = 0.00099). For the probe microphone, the most substantial improvement was at 397 Hz (9.8 dB, *p* = 0.00038), with significant improvements also at 250 Hz (5.1 dB, *p* = 0.01611), 315 Hz (9.0 dB, *p* = 0.00064), 500 Hz (6.4 dB, *p* = 0.01845), and 1000 Hz (5.7 dB, *p* = 0.02009).

**Table 3:** Mean hearing threshold comparison and statistical significance for the accelerometer (left table) and the probe microphone (right table). Each table presents the mean hearing thresholds at specific frequencies with and without crosstalk cancellation. The corresponding *p*-values are provided to indicate the statistical significance of the observed improvements in hearing thresholds due to crosstalk cancellation.

Accelerometer				Probe Microphone			
Freq. (Hz)	Mean (sd) hear. thresh. (dB re. 1V_RMS_)		*p-value*	Freq. (Hz)	Mean (SD) Hear. Thresh. (dB re. 1V_RMS_)		*p-value*
	without x-tk. canc.	with x-tk. canc.			without x-tk. canc.	with x-tk. canc.	
250	−36.1 (5.1)	−41.6 (5.5)	0.03575*	250	−34.2 (5.6)	−39.3 (5.6)	0.01611*
315	−44.0 (5.0)	−53.5 (5.7)	0.00018⁎⁎⁎	315	−38.3 (5.6)	−47.3 (8.1)	0.00064⁎⁎⁎
397	−42.5 (5.9)	−48.6 (6.3)	0.00099⁎⁎⁎	397	−40.0 (5.8)	−49.8 (6.4)	0.00038⁎⁎⁎
500	−42.4 (5.7)	−44.4 (4.8)	0.06259	500	−39.4 (6.4)	−45.8 (7.2)	0.01845*
630	−43.0 (8.0)	−44.4 (5.7)	0.26818	630	−44.1 (6.1)	−44.8 (9.5)	0.78811
794	−50.0 (4.2)	−53.4 (7.0)	0.11172	794	−50.8 (9.4)	−51.9 (10.1)	0.62868
1000	−48.6 (6.1)	−50.1 (6.2)	0.59115	1000	−46.5 (6.7)	−52.2 (7.8)	0.02009*

⁎ *p* < 0.05; **p < 0.01;.

⁎⁎⁎ *p* < 0.001.

In light of our observations regarding hearing threshold improvements (as seen in the bottom panels of Fig. 13(a) and (b)), our findings suggest that although the cancellation was designed for specific sites—the ear canal, monitored with a probe microphone, and the mastoid, monitored via an accelerometer—its effects seem to extend beyond these sensor locations. This supports the hypothesis of a “zone of quiet” potentially influencing areas like the cochlea. Yet, due to complexities such as the occlusion effect in the ear canal, it is challenging to definitively conclude the direct impact of this “zone” on the cochlea. Furthermore, the effectiveness of this process appears to be frequency-dependant. Our data indicates that lower frequencies result in more noticeable improvements in hearing thresholds. This trend can be attributed to the size of the wavelength: the “zone of quiet” covers a larger area at lower frequencies, in line with established theories in active noise control [13].

**Fig. 14:**
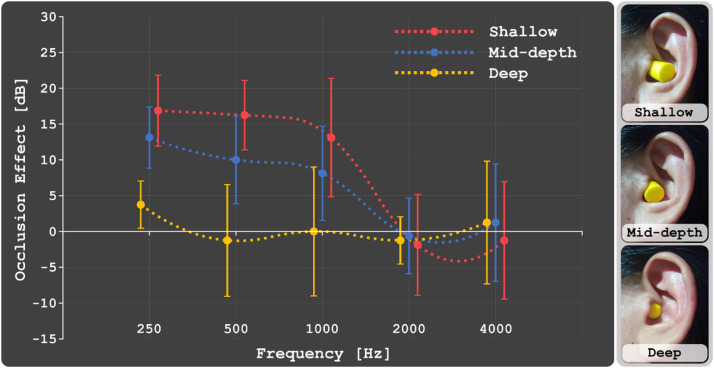
Average occlusion effect measured across audiometric frequencies (250, 500, 1000, 2000, and 4000 Hz). The effect is defined as the difference in BC hearing thresholds with and without earplug occlusion. The graph represents three earplug insertion depths: Shallow (25 %, ∼6 mm), Mid-depth (50 %, ∼12 mm), and Deep (75–100 %, 18–24 mm). Accompanying images provide a visual representation of each insertion depth.

In our experiment with normally-hearing subjects, the potential influence of the occlusion effect, even with deep earplug insertion, introduces an element of ambiguity. Fig. 14 presents the results of a supplementary experiment that quantified the occlusion effect at various earplug insertion depths. These measurements, taken at audiometric frequencies (250, 500, 1000, 2000, and 4000 Hz), utilized the Resonance's R17A-BC audiometer. The occlusion effect is defined as the difference in BC hearing thresholds with and without earplug occlusion [29]. We evaluated three insertion depths: Shallow (25 %, approximately 6 mm), Mid-depth (50 %, approximately 12 mm), and Deep (75–100 %, 18–24 mm). The accompanying images in Fig. 14 visually represent each insertion depth. The data indicates that even at a 75–100 % deep insertion, the occlusion effect is not entirely eliminated, though it is reduced compared to the shallow condition. This observation leads us to consider the possibility that the crosstalk cancellation we observed in our experiments might be occurring both at the cochlear and ear canal levels, with the latter potentially intensified by the occlusion effect. This dual possibility underscores the importance of future studies involving individuals with conductive hearing loss to more definitively determine the extent of crosstalk cancellation at the cochlear level.

Upon closer examination of each frequency, we found that the improvement in hearing thresholds when the cancellation site was on the mastoid—a region behind the ear—was limited to frequencies up to 397 Hz. This area may be considered somewhat distant in comparison to the ear canal. On the other hand, when we used a probe microphone in the ear canal, the improvement extended up to 1000 Hz, though it significantly diminished at 630 Hz (0.7 dB) and 794 Hz (1.1 dB). At these two frequencies, some participants actually experienced an increase in hearing thresholds with the addition of anti-noise, suggesting that the anti-noise signal may have inadvertently amplified the crosstalk sound—a result that contradicts our expectations. Such amplification could be due to variations in phase relationships caused by the distance between the sensor and the cochlea in the inner ear.

These results suggest that crosstalk cancellation at the cochlea can potentially be achieved by cancelling crosstalk sounds at sensor locations. However, this method's effectiveness may diminish at higher frequencies where the designed phase mismatch at the sensor location does not yield optimal cancellation at the cochlea. Further research is necessary to ascertain whether crosstalk cancellation in bone conduction can genuinely enhance sound localization and improve overall hearing experiences. As our proposed approach demonstrates efficacy mainly at low frequencies, it might be beneficial to integrate this method with a psychoacoustic-based crosstalk cancellation method, such as the “two-BT” method proposed by McLeod and Culling [11], to broaden the frequency range. Furthermore, it would be worthwhile to assess this method's performance in a larger sample of participants, including individuals with conductive hearing loss, to pinpoint areas for potential enhancement.

## Conclusion

In this study, we proposed and evaluated a method for crosstalk cancellation through bone conduction. Our approach assumes that the cancellation of crosstalk sounds in the ear canal, as measured by a probe microphone, or on the mastoid, as detected by an accelerometer, can effectively reach the cochlea in the inner ear. By employing the FxLMS adaptive algorithm, we demonstrated that sound signals from two audiometric bone transducers could be effectively neutralized at the sensor locations. We also found that participants’ hearing thresholds were improved with the application of crosstalk cancellation. However, the effectiveness of our method was primarily observed at low frequencies. At higher frequencies, due to the distance between the cochlea and the cancellation site, the designed phase mismatch at the sensor location did not result in optimal cancellation at the cochlea. This led to a suboptimal phase relationship between the crosstalk sound and the anti-crosstalk signal, limiting the effectiveness of the cancellation. Future work could consider combining our current approach with a psychoacoustic-based method, which could potentially address the phase mismatch issue and lead to more effective cancellation at the cochlea across a wider frequency range. Additionally, testing our method on people with conductive hearing loss is important. This can help us see where our approach might need improvement and find ways to make it work better.

## Ethics statements

This work involved human subjects. Written informed consent was collected from all participants utilizing a protocol (No. 29–13) approved by the Institutional Review Board of Life Science Research of Chiba University, Chiba, Japan.

## Declaration of Competing Interest

The authors declare that they have no known competing financial interests or personal relationships that could have appeared to influence the work reported in this paper.

## Data Availability

Data will be made available on request.
